# Beyond donor eligibility: nutritional and lifestyle heterogeneity and implications for long-term donor health preservation

**DOI:** 10.3389/fnut.2026.1903623

**Published:** 2026-09-03

**Authors:** Elisa Mazza, Samantha Maurotti, Yvelise Ferro, Francesca Rita Noto, Carmelo Pujia, Nancy Paone, Alessia Sestito, Marta Moraca, Ilda Fatima Martino, Arturo Pujia, Tiziana Montalcini

**Affiliations:** 1 Department of Clinical and Experimental Medicine, University Magna Græcia, Catanzaro, Italy; 2 Department of Medical and Surgical Sciences, University Magna Græcia, Catanzaro, Italy; 3 Clinical Nutrition Unit, AOU “Renato Dulbecco”, Catanzaro, Italy; 4 Research Center for the Prevention and Treatment of Metabolic Diseases, University “Magna Græcia”, Catanzaro, Italy

**Keywords:** BMI, dietary patterns, donation, lifestyle, nutrition

## Abstract

**Background:**

Blood donor eligibility is essential for donation safety, but it may not fully capture nutritional, metabolic, and lifestyle-related vulnerabilities that can affect donor health over time. As transfusion systems increasingly rely on regular donors, identifying modifiable factors that may support long-term donor health and retention is becoming increasingly relevant. This study aimed to characterize nutritional and lifestyle heterogeneity, peri-donation dietary behaviors, and donation-related outcomes in adult blood donors.

**Methods:**

This cross-sectional survey used a structured online questionnaire assessing anthropometric characteristics, lifestyle factors, habitual dietary intake, nutrition-related awareness, peri-donation dietary behaviors, self-reported history of iron deficiency, and post-donation symptoms. Dietary profiles were identified using principal component analysis followed by k-means clustering. Multivariable logistic regression models were used to explore factors associated with iron deficiency history and post-donation symptoms.

**Results:**

The study included 698 adult blood donors. Overweight and obesity affected nearly half of the cohort and were more frequent among men and older donors, whereas women more frequently reported iron deficiency history and post-donation symptoms. Three dietary profiles were identified: Western/ultra-processed, Mediterranean-like, and mixed beverage-oriented. Younger donors were more frequently represented in less favorable dietary and lifestyle profiles, whereas older donors showed more favorable dietary habits despite a higher prevalence of overweight/obesity. In multivariable analyses, female sex was independently associated with iron deficiency history (OR 4.62, 95% CI 3.20–6.68; *p* < 0.001) and post-donation symptoms (OR 3.07, 95% CI 2.09–4.52; *p* < 0.001), while increasing age was inversely associated with post-donation symptoms (OR 0.73 per 10-year increase, 95% CI 0.64–0.84; *p* < 0.001). Dietary pattern membership was not independently associated with either outcome. Peri-donation dietary changes were reported more frequently before donation than after donation.

**Discussion:**

These findings suggest that adult blood donors represent a nutritionally and metabolically heterogeneous population despite donation eligibility. These findings support the potential relevance of structured nutritional counseling, lifestyle-oriented prevention, and recovery-focused recommendations within donor care pathways, although their impact on long-term donor health, continued eligibility, and donor retention should be evaluated in future prospective and interventional studies.

## 1 Introduction

Blood donation is an essential component of modern healthcare systems and remains the only irreplaceable source of blood and blood components for transfusion therapy. Despite major technological advances in medicine, no artificial substitute fully replicates the complex biological and functional properties of human blood, making voluntary donation indispensable for the management of trauma, surgery, hematologic disorders, cancer, and obstetric emergencies (1, 2). Globally, more than 118 million blood units are collected annually, yet donor availability remains insufficient in many healthcare systems (2). Maintaining an adequate and stable donor pool represents a major challenge for transfusion services, not only because of the difficulty in recruiting new donors, but also because donor discontinuation progressively occurs over time even among regular donors (3). Previous studies have shown that donor attrition is frequently associated with adverse donation experiences, temporary deferral, and health-related conditions (4). As modern transfusion systems increasingly depend on regular donors, preserving donor health has become increasingly relevant for transfusion sustainability (5).

Eligibility criteria for donation are designed to protect both donors and recipients and contribute to the well-recognized “healthy donor effect,” whereby donors generally display lower morbidity and more favorable health profiles compared with the general population (6, 7). However, eligibility screening primarily excludes overt disease and may not fully capture subclinical metabolic or behavioral vulnerability. Consequently, donor populations may still include individuals characterized by excess body weight, sedentary lifestyles, or unfavorable dietary habits despite continued donation eligibility. This aspect is particularly relevant considering the increasing prevalence of obesity and lifestyle-related metabolic disorders in the general population, conditions that may remain clinically silent while progressively affecting long-term donor health and eligibility.

Although cardiovascular and metabolic risk factors in blood donors have been partially explored (8, 9), considerably less attention has been directed toward dietary behaviors, peri-donation nutritional practices, and nutrition-related awareness. Dietary habits may influence iron homeostasis, procedural tolerance, post-donation recovery, and overall metabolic risk profiles (10, 11). Whole blood donation induces acute hematologic changes and substantial loss of iron stores (12–15), while repeated donations may contribute to progressive iron depletion, fatigue, reduced functional capacity, and temporary deferral from donation, particularly among women and younger donors (16–18). In addition, mild adverse events such as dizziness and vasovagal reactions are relatively common and may be influenced by hydration status and overall nutritional condition (19–21). Nevertheless, real-world evidence characterizing donors’ habitual dietary patterns and nutrition-related awareness remains limited, and recent surveys indicate insufficient knowledge regarding appropriate nutritional practices before and after donation (22). Taken together, these observations support the need for preventive donor-centered strategies aimed at improving donor health and long-term transfusion sustainability (11, 23–26).

Therefore, the present study aimed to investigate nutritional and lifestyle heterogeneity in a large cohort of adult blood donors by characterizing dietary habits, nutrition knowledge, and peri-donation dietary behaviors, while also exploring factors associated with self-reported history of iron deficiency and post-donation symptoms. More broadly, the study sought to examine whether donor populations commonly perceived as uniformly healthy may in fact include distinct profiles of nutritional and behavioral vulnerability, with potential implications for long-term donor health preservation.

## 2 Materials and methods

### 2.1 Study design and population

This observational cross-sectional study investigated dietary habits, nutrition knowledge, and peri-donation dietary behaviors among adult blood donors. The survey was promoted by the Clinical Nutrition Unit of the University “Magna Græcia” of Catanzaro and administered between 23 April 2025 and 6 September 2025 using an anonymous web-based questionnaire developed and distributed through Google Forms (Google LLC, Mountain View, CA, USA). Eligible participants were men and women aged ≥ 18 years who had donated whole blood at least once in their lifetime. Participation was voluntary and uncompensated. No exclusion criteria were applied other than age below 18 years or absence of previous donation experience. Electronic informed consent was obtained prior to participation. The study complied with the Declaration of Helsinki and was approved by the Local Ethics Committee of the Calabria Region (approval code 190/2025). Recruitment was conducted through institutional mailing lists and social media platforms (Facebook, WhatsApp, and Instagram) to reach both first-time and regular donors. Recruitment strategies were designed to maximize participation across donors with different demographic and donation profiles.

### 2.2 Questionnaire development and data collection

A multidisciplinary team including clinical nutritionists and transfusion medicine specialists developed a structured online questionnaire to assess nutritional behaviors, donation-related practices, and nutrition-related awareness among blood donors. The survey collected self-reported information across multiple domains, including sociodemographic characteristics, anthropometric measures, lifestyle factors, habitual dietary intake, nutrition-related awareness, and peri-donation dietary behaviors (Supplementary Table 1).

Item selection was informed by previously published instruments assessing nutrition knowledge and dietary behaviors in adult populations (27, 28), and adapted to the specific context of blood donation.

To support content validity, the questionnaire was reviewed by a multidisciplinary team with expertise in clinical nutrition, dietetics, and transfusion medicine, who assessed item relevance, clarity, comprehensiveness, and appropriateness for the donor population.

The questionnaire was pilot-tested in 20 adult volunteers to improve clarity and readability prior to dissemination. Minor wording adjustments were implemented to improve readability.

Sociodemographic data included age, sex, marital status, educational and occupational status, and the perceived influence of work on eating behaviors. Health- and lifestyle-related information comprised smoking status, physical activity, history of iron deficiency, chronic diseases, and regular medication use. Participants also reported body weight and height for BMI calculation.

Participants reported whether they had ever experienced iron deficiency or had previously been informed by a healthcare professional of reduced iron stores or low iron levels. For descriptive analyses, responses were retained in their original four-category format (“never,” “do not know,” “recent self-reported iron deficiency,” and “previous self-reported iron deficiency”). For logistic regression analyses, participants reporting either previous or recent iron deficiency were grouped into a binary outcome variable defined as “self-reported history of iron deficiency”; participants answering “do not know” were excluded from models involving this outcome. Habitual dietary intake was assessed through semi-quantitative food frequency–based questions investigating the consumption of major food groups, including cereals, fruit and vegetables, legumes, animal protein sources, processed foods, sugar-sweetened beverages, and alcoholic drinks. Portion frequencies were recorded using standardized response categories to ensure comparability. Participants were additionally asked whether they followed specific dietary patterns (e.g., vegetarian, vegan, pescatarian, or omnivorous).

The survey was designed to characterize overall dietary behaviors and relative differences across participants rather than estimate absolute nutrient intake.

Peri-donation dietary behaviors were explored by asking participants whether their dietary habits changed during the week preceding and the week following donation compared with their usual habits.

Post-donation symptoms were assessed through self-reported items including fatigue, dizziness, and other self-reported donation-related symptoms. Information on menstrual blood loss, menopausal status, hormonal contraceptive use, and menopausal hormone therapy was not collected.

Nutrition-related awareness was assessed through six targeted questions exploring hydration, meal composition, micronutrients involved in erythropoiesis, and dietary factors affecting iron absorption.

Items were analyzed individually, and the proportion of correct responses was calculated for each question. As an additional reliability check, the internal consistency of the six nutrition knowledge items was assessed. The questionnaire primarily consisted of closed-ended multiple-choice items. Completion time was approximately 8–10 min.

Given the exploratory nature of the study, formal construct validation of the overall questionnaire was not performed. However, questionnaire items were informed by previous literature and reviewed by healthcare professionals to improve content relevance and readability. All variables, including anthropometric data, history of iron deficiency, and post-donation symptoms, were self-reported.

### 2.3 Dietary assessment and anthropometric variables

Food frequency responses were treated as ordinal variables and coded numerically (0 = none/never; 1 = one portion; 2 = two portions; 3 = three or more portions) to approximate consumption frequency. These scores were used for relative comparisons across participants and not as estimates of absolute dietary intake.

Body weight and height were self-reported, and Body Mass Index (BMI) was calculated as weight (kg)/height (m^2^). Implausible anthropometric values (height < 120 or > 220 cm; weight < 30 or > 200 kg) were excluded prior to BMI calculation. Consequently, analyses involving anthropometric variables were conducted on complete cases only (*n* = 694).

### 2.4 Statistical analysis

Continuous variables are presented as mean ± standard deviation (SD), whereas categorical variables are expressed as frequencies and percentages. Between-group differences for continuous variables were assessed using Student’s independent-samples *t*-test when comparing two groups and one-way analysis of variance (ANOVA) when comparing more than two groups. For variables showing significant overall differences across dietary clusters, exploratory *post-hoc* pairwise comparisons were performed. Tukey’s *post-hoc* test was used for continuous variables, whereas Bonferroni-adjusted pairwise comparisons were used for categorical variables. Associations between categorical variables, including sex differences in questionnaire responses and nutrition knowledge items, were evaluated using Pearson’s chi-square (χ^2^) tests. Nutrition knowledge questions were analyzed at the item level as independent categorical variables. Internal consistency of the six dichotomized nutrition knowledge items was assessed using Cronbach’s alpha, which is equivalent to the Kuder–Richardson Formula 20 for binary items. Analyses were additionally stratified by sex and age group. To identify overall dietary patterns, standardized food-frequency variables were subjected to principal component analysis (PCA) based on the correlation matrix.

Sampling adequacy and factorability were assessed using the Kaiser–Meyer–Olkin (KMO) statistic and Bartlett’s test of sphericity. The number of retained components was guided by scree plot inspection, interpretability, and parsimony. Varimax rotation was applied to enhance interpretability. Factor loadings ≥ | 0.30| were considered meaningful contributors to each dietary pattern, and the rotated factor loadings are presented in Supplementary Table 4. Component scores were then used as input variables for k-means clustering to classify participants into mutually exclusive dietary pattern groups. A three-cluster solution was selected because it was clinically interpretable and consistent with the three retained PCA-derived dietary profiles. To improve robustness, k-means clustering was performed using multiple random initializations. The primary analytical outcomes were self-reported history of iron deficiency and post-donation symptoms. Both outcomes were based on self-reported questionnaire items. For analysis, both were dichotomized and treated as binary variables. Multivariable logistic regression models were used to examine associations between participant characteristics and these outcomes, adjusting simultaneously for age, sex, BMI, smoking status, physical activity, and dietary pattern membership. Results are reported as odds ratios (ORs) with 95% confidence intervals (CIs). An *a priori* sample size calculation, based on an expected prevalence of post-donation adverse effects of approximately 36% (29), indicated a minimum of 553 participants (95% confidence level; ± 4% precision). The final sample (*n* = 698) exceeded this requirement. Missing data were minimal and handled using complete-case analysis. All tests were two-sided, with statistical significance set at *p* < 0.05. Given the exploratory nature of the study, no formal adjustment was applied across the full set of analyses. However, *post-hoc* pairwise comparisons following significant overall differences across dietary clusters were adjusted as described above. Statistical analyses were performed using Python-based computational workflows.

## 3 Results

### 3.1 Characteristics of the study population

The baseline characteristics of the study population are presented in Table 1. The study included 698 blood donors (297 men, 42.6%; 401 women, 57.4%), with a mean age of 35.4 ± 14.0 years.

**TABLE 1:** Baseline characteristics of the blood donor population (overall and stratified by sex).

Baseline characteristics	Total	Male	Female	*p*-value
Demographic and anthropometric data				
Sex, *n* (%)	698	297 (42.6)	401 (57.4)	
Age (years)	35.41 ± 14.01	38.75 ± 14.21	32.94 ± 13.35	< 0.001
Body weight (kg)	72.55 ± 15.04	81.30 ± 12.58	66.08 ± 13.35	< 0.001
Height (m)	1.69 ± 0.09	1.76 ± 0.07	1.64 ± 0.07	< 0.001
BMI (kg/m^2^)	25.35 ± 4.61	26.22 ± 3.61	24.70 ± 5.15	< 0.001
BMI categories, n (%)				
Normal weight	356 (51.3)	117 (39.5)	239 (60.1)	< 0.001
Underweight	8 (1.2)	0 (0.0)	8 (2.0)	
Overweight	250 (36.0)	142 (48.0)	108 (27.1)	
Obese	80 (11.5)	37 (12.5)	43 (10.8)	
Marital status, n (%)				
Married or cohabiting	398 (57.0)	181 (60.9)	217 (54.1)	0.219
Separated/divorced	19 (2.7)	6 (2.0)	13 (3.2)	
Single	276 (39.5)	109 (36.7)	167 (41.6)	
Widowed	5 (0.7)	1 (0.3)	4 (1.0)	
Socioeconomic factors and donation status				
Occupation, n (%)				
Unemployed	39 (5.6)	7 (2.4)	32 (8.0)	< 0.001
Office worker	207 (29.7)	107 (36.0)	100 (24.9)	
Manual/office worker	117 (16.8)	73 (24.6)	44 (11.0)	
Retired	10 (1.4)	7 (2.4)	3 (0.7)	
Healthcare professional (physician, nurse, etc.)	83 (11.9)	26 (8.8)	57 (14.2)	
Student	242 (34.7)	77 (25.9)	165 (41.1)	
Work influence on dietary habits, n (%)				
No, follows a balanced diet	370 (53.0)	156 (52.5)	214 (53.4)	0.201
Yes, irregular meal timing	144 (20.6)	54 (18.2)	90 (22.4)	
Yes, eats outside home/quick meals	184 (26.4)	87 (29.3)	97 (24.2)	
Health status and lifestyle factors				
Smoking status, n (%)				
Yes	192 (27.5)	91 (30.6)	101 (25.2)	0.131
No	506 (72.5)	206 (69.4)	300 (74.8)	
History of iron deficiency, n (%)				
Never	412 (59.0)	236 (79.5)	176 (43.9)	< 0.001
Do not know	42 (6.0)	13 (4.4)	29 (7.2)	
Self-reported recent iron deficiency	72 (10.3)	11 (3.7)	61 (15.2)	
Self-reported previous iron deficiency	172 (24.6)	37 (12.5)	135 (33.7)	
Physical activity frequency, n (%)				
None	207 (29.7)	76 (25.6)	131 (32.7)	0.003
Occasionally	222 (31.8)	85 (28.6)	137 (34.2)	
Regularly	269 (38.5)	136 (45.8)	133 (33.2)	
Comorbidities, n (%)				
Yes ( ≥ 1 condition)	188 (26.9)	67 (22.6)	121 (30.2)	0.031
No (none)	510 (73.1)	230 (77.4)	280 (69.8)	
Regular medication use, n (%)				
No	387 (55.4)	185 (62.3)	202 (50.4)	< 0.001
Yes, for chronic diseases	104 (14.9)	51 (17.2)	53 (13.2)	
Yes, occasionally	207 (29.7)	61 (20.5)	146 (36.4)	
Dietary Pattern, n (%)				
No specific diet (omnivorous)	633 (90.7)	280 (94.3)	353 (88.0)	0.066
Other	39 (5.6)	11 (3.7)	28 (7.0)	
Pescatarian	11 (1.6)	2 (0.7)	9 (2.2)	
Vegan	3 (0.4)	0 (0.0)	3 (0.7)	
Vegetarian	10 (1.4)	4 (1.3)	6 (1.5)	
Influence of blood donation on eating habits, n (%)				
No, eat normally	498 (71.3)	222 (74.7)	276 (68.8)	0.196
No, never considered it	159 (22.8)	58 (19.5)	101 (25.2)	
Yes	39 (5.6)	17 (5.7)	22 (5.5)	

Continuous variables are presented as mean ± SD and compared using an independent-samples *t*-test. Categorical variables are presented as *n* (%) and compared using the χ^2^ test.

Men were significantly older and had higher body weight, height, and BMI than women (all *p* < 0.001), with mean BMI values of 26.22 ± 3.61 kg/m^2^ in men and 24.70 ± 5.15 kg/m^2^ in women.

BMI category distribution differed significantly by sex (*p* < 0.001). Women were more frequently classified as normal weight compared with men (60.1% vs. 39.5%), whereas men had a higher prevalence of overweight (48.0% vs. 27.1%). The prevalence of obesity was comparable between sexes.

Marital status did not differ significantly between men and women (*p* = 0.219), whereas occupational status differed significantly (*p* < 0.001), with a higher proportion of students among women and a higher proportion of office and manual workers among men.

No significant sex differences were observed in work-related dietary habits (*p* = 0.201) or smoking status (*p* = 0.131). In contrast, a self-reported history of iron deficiency was significantly more prevalent among women than men (*p* < 0.001), including both self-reported previous and recently reported iron deficiency. Physical activity levels differed significantly between sexes (*p* = 0.003), with men more frequently reporting regular physical activity and women more frequently reporting no physical activity. The prevalence of comorbidities was higher among women (30.2% vs. 22.6%; *p* = 0.031), and regular medication use also differed significantly by sex (*p* < 0.001), with women more frequently reporting occasional medication use. Self-reported dietary habits were predominantly omnivorous (90.7%) and did not differ significantly between sexes (*p* = 0.066). Similarly, eating habits related to blood donation were comparable between men and women (*p* = 0.196).

### 3.2 Age-related differences

Age-stratified analyses (Supplementary Table 2) showed significant differences across age groups. BMI increased progressively with advancing age (*p* < 0.001), accompanied by a higher prevalence of overweight and obesity (*p* < 0.001). Regular physical activity decreased significantly with age (*p* < 0.001), whereas post-donation symptoms were more frequently reported among younger donors and declined with increasing age (*p* < 0.001).

The proportion of female participants decreased significantly with increasing age (*p* < 0.001). In contrast, no significant differences across age groups were observed for smoking status (*p* = 0.126) or self-reported history of iron deficiency (*p* = 0.590).

### 3.3 Habitual dietary intake

Habitual dietary intake is summarized in Table 2. Overall, donors reported moderate weekly consumption of legumes, dairy products, meat, and fish, and a daily intake of approximately 1–2 portions of fruit and vegetables.

**TABLE 2:** Habitual dietary intake of the blood donor population (overall and stratified by sex).

Food group	Total	Male	Female	*p*-value
Legumes (week)	1.37 ± 0.84	1.31 ± 0.82	1.42 ± 0.85	0.082
Cheese (week)	1.63 ± 0.90	1.70 ± 0.92	1.58 ± 0.88	0.091
Fresh meat (week)	1.86 ± 0.89	2.05 ± 0.86	1.72 ± 0.88	< 0.001
Processed meat (week)	1.20 ± 0.89	1.36 ± 0.92	1.08 ± 0.85	< 0.001
Eggs (week)	1.41 ± 0.78	1.46 ± 0.80	1.37 ± 0.76	0.124
Fish (week)	1.59 ± 0.81	1.62 ± 0.83	1.56 ± 0.80	0.324
Pizza (week)	1.42 ± 0.90	1.55 ± 0.93	1.33 ± 0.87	0.002
Fried foods (week)	1.37 ± 0.87	1.47 ± 0.89	1.29 ± 0.85	0.006
Ready meals (week)	1.40 ± 0.87	1.50 ± 0.89	1.32 ± 0.85	0.010
Fruit (day)	1.66 ± 0.95	1.55 ± 0.92	1.74 ± 0.96	0.009
Vegetables (day)	1.83 ± 0.95	1.72 ± 0.92	1.91 ± 0.96	0.011
Milk/yogurt (day)	1.47 ± 0.91	1.44 ± 0.90	1.49 ± 0.92	0.448
Olive oil (day)	1.60 ± 0.82	1.58 ± 0.81	1.62 ± 0.83	0.536
Sugary drinks (day)	1.36 ± 0.86	1.48 ± 0.88	1.27 ± 0.84	0.001
Wine (week)	1.42 ± 0.93	1.63 ± 0.95	1.27 ± 0.89	< 0.001
Beer (week)	1.38 ± 0.86	1.59 ± 0.88	1.22 ± 0.82	< 0.001

Food frequency responses were coded as 0–3 (0 = none/never; 1 = one portion; 2 = two portions; 3 = three or more portions). Values are expressed as mean frequency scores on a 0–3 scale. Higher scores indicate higher consumption frequency. *p*-values were obtained from independent-samples *t-*tests.

Several sex differences were observed. Compared with women, men reported significantly higher weekly consumption of fresh meat and processed meat (both *p* < 0.001), as well as greater intake of pizza, fried foods, and ready meals (all *p* ≤ 0.010). Men also reported significantly higher consumption of sugar-sweetened beverages and alcoholic drinks, including wine and beer (all *p* ≤ 0.001).

Conversely, women reported significantly higher daily consumption of fruit (*p* = 0.009) and vegetables (*p* = 0.011). No significant sex differences were observed in the consumption of legumes, cheese, eggs, fish, milk/yogurt, or olive oil (all *p* > 0.05).

Age-stratified analyses (Supplementary Table 3) showed progressively greater adherence to Mediterranean-like dietary habits with increasing age, characterized by higher consumption of fish, fruit, vegetables, and olive oil, together with lower intake of pizza, fried foods, ready meals, and sugar-sweetened beverages.

### 3.4 Nutrition knowledge

**FIGURE 1:**
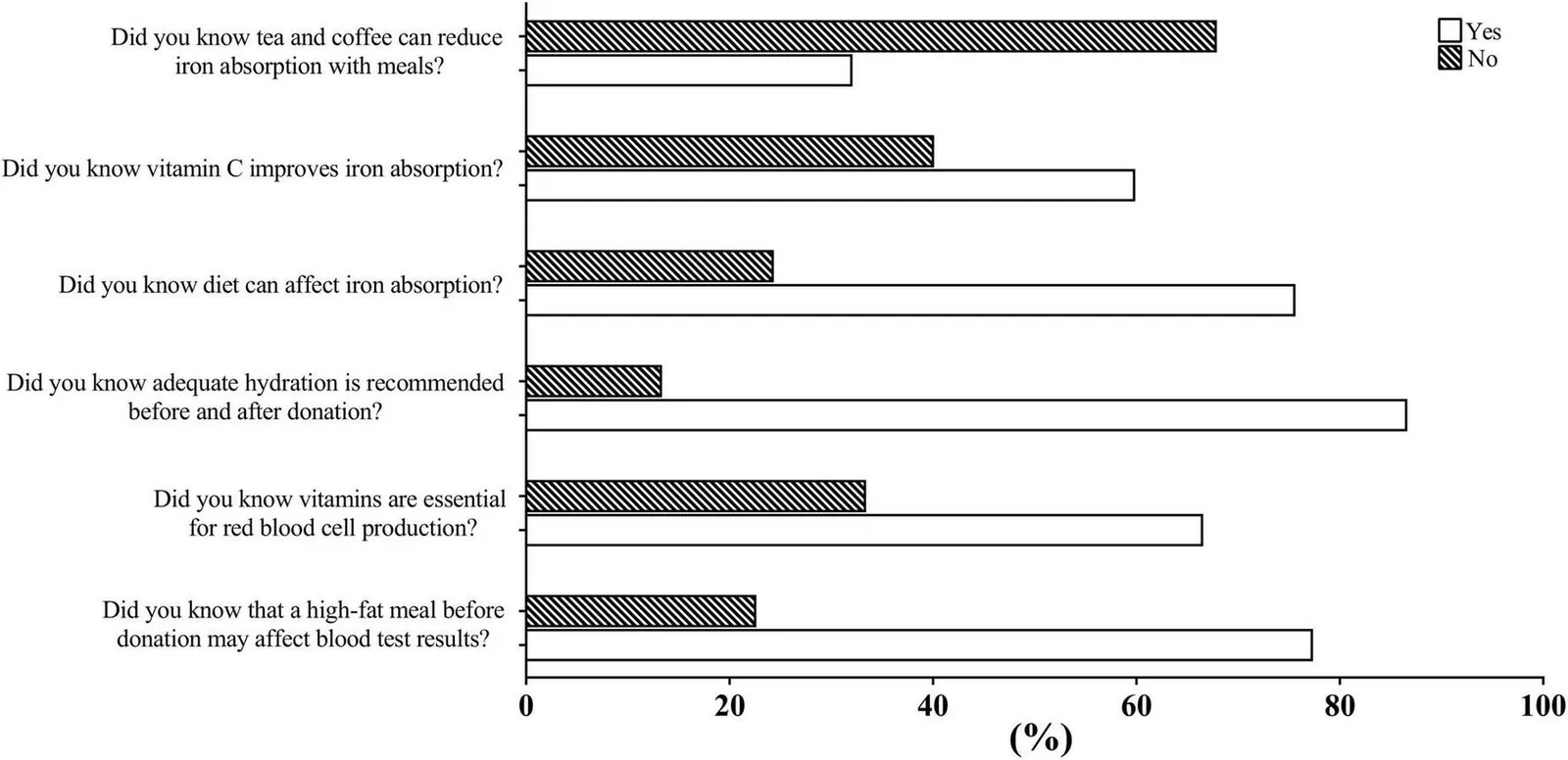
Nutrition-related knowledge among blood donors.

Nutrition knowledge results are presented in Figure 1. The six nutrition knowledge items showed acceptable internal consistency (Cronbach’s α = 0.70; *n* = 693 complete cases). Awareness was highest regarding adequate hydration before and after donation, whereas the lowest proportion of correct responses concerned the inhibitory effect of tea and coffee on iron absorption. Intermediate levels of awareness were observed for the remaining items. No significant sex differences were observed for any knowledge item (all *p* > 0.05).

### 3.5 Dietary patterns

Principal component analysis with Varimax rotation followed by k-means clustering identified three distinct dietary profiles (Table 3 and Supplementary Table 4): a Western/ultra-processed profile, characterized by higher intake of processed meat, fried foods, ready meals, pizza, and sugar-sweetened beverages; a Mediterranean-like profile, characterized by higher consumption of fruit, vegetables, legumes, fish, and olive oil; and a mixed beverage-oriented profile, mainly characterized by wine and beer consumption. The correlation matrix was suitable for PCA, as indicated by a KMO value of 0.72 and a significant Bartlett’s test of sphericity (χ^2^ = 1861.36, df = 120, *p* < 0.001). The three retained components explained 17.3%, 14.4%, and 9.0% of the variance, respectively, accounting for 40.7% of the total variance.

**TABLE 3:** Dietary clusters and clinical/lifestyle correlates.

Variable	Western/ultra-processed-like (*n* = 195)	Mediterranean-like (*n* = 139)	Mixed beverage-oriented pattern (*n* = 364)	*p*-value
Age, years	32.4 ± 12.5	35.5 ± 14.2	37.0 ± 14.5	0.001
Female, *n* (%)	102 (52.3)	80 (57.6)	219 (60.2)	0.201
BMI, kg/m^2^	25.7 ± 4.8	25.1 ± 3.8	25.2 ± 4.2	0.269
Overweight/obesity (BMI ≥ 25), *n* (%)	91 (46.7)	70 (50.4)	169 (46.4)	0.717
Smoking, *n* (%)	75 (38.5)	40 (28.8)	77 (21.2)	< 0.001
Regular physical activity, *n* (%)	58 (29.7)	54 (38.8)	157 (43.1)	0.002
Any history of iron deficiency, *n* (%)	63 (32.3)	49 (35.3)	132 (36.3)	0.561
Post-donation symptoms, *n* (%)	62 (31.8)	51 (36.7)	129 (35.4)	0.509

Overall *p*-values were obtained from ANOVA or χ^2^ tests, as appropriate. For variables showing significant overall differences across dietary clusters, exploratory *post-hoc* pairwise comparisons were performed using Tukey’s test for continuous variables and Bonferroni-adjusted pairwise comparisons for categorical variables.

Participants differed significantly across dietary clusters for age, smoking status, and regular physical activity (all omnibus *p* < 0.01), whereas no significant differences were observed for BMI, overweight/obesity prevalence, self-reported history of iron deficiency, or post-donation symptoms. *Post-hoc* pairwise analyses showed that participants in the Western/ultra-processed cluster were younger than those in the mixed beverage-oriented cluster (Tukey *p* < 0.001), had a higher prevalence of smoking (Bonferroni-adjusted *p* < 0.001), and had a lower prevalence of regular physical activity (Bonferroni-adjusted *p* = 0.006). Other pairwise comparisons were not significant after correction.

Although dietary and lifestyle profiles differed substantially across clusters, these differences were not accompanied by significant differences in self-reported history of iron deficiency or post-donation symptoms.

### 3.6 Sex- and age-stratified lifestyle profiles across dietary patterns

Sex- and age-stratified analyses across dietary patterns are presented in Table 4. Among both women and men, BMI and the prevalence of overweight/obesity were generally higher in donors aged ≥ 35 years across dietary profiles. Any physical activity varied across dietary patterns and was consistently lower in the Western/ultra-processed profile than in the Mediterranean-like and mixed profiles, particularly among women aged ≥ 35 years.

**TABLE 4:** Sex- and age-stratified anthropometric and lifestyle characteristics across dietary patterns.

Men			
Variable	Western/UPF-like (*n* = 47)	Mediterranean-like (*n* = 26)	Mixed (*n* = 60)
Men < 35 years			
BMI, kg/m^2^	26.0 ± 3.3	25.2 ± 3.4	24.8 ± 3.1
Overweight/obesity, *n* (%)	20 (42.6)	12 (46.2)	24 (40.0)
Any physical activity, *n* (%)	33 (70.2)	24 (92.3)	55 (91.7)
**Variable**	**Western/UPF-like (*n* = 46)**	**Mediterranean-like (n = 34**)	**Mixed (*n* = 84)**
Men ≥ 35 years			
BMI, kg/m^2^	27.1 ± 3.5	27.1 ± 3.7	27.0 ± 3.3
Overweight/obesity, *n* (%)	24 (53.3)	26 (76.5)	54 (63.5)
Any physical activity, *n* (%)	23 (51.1)	25 (73.5)	61 (71.8)
**Women**			
**Variable**	**Western/UPF-like (*n* = 71)**	**Mediterranean-like (*n* = 56)**	**Mixed (*n* = 133)**
Women <35 years			
BMI, kg/m^2^	24.7 ± 4.3	23.5 ± 4.5	24.0 ± 4.1
Overweight/obesity, *n* (%)	22 (31.0)	15 (26.8)	37 (27.8)
Any physical activity, *n* (%)	40 (56.3)	44 (78.6)	100 (75.2)
**Variable**	**Western/UPF-like (*n* = 31)**	**Mediterranean-like (*n* = 23)**	**Mixed (*n* = 87)**
Women ≥ 35 years			
BMI, kg/m^2^	26.9 ± 4.5	24.8 ± 5.0	25.8 ± 4.2
Overweight/obesity, *n* (%)	14 (45.2)	8 (34.8)	39 (44.8)
Any physical activity, *n* (%)	9 (29.0)	14 (60.9)	63 (72.4)

BMI values are presented as mean ± SD. Overweight/obesity and any physical activity are presented as *n* (%). “Any physical activity” includes participants reporting occasional or regular physical activity. Percentages refer to each sex-, age-, and dietary pattern-specific subgroup. UPF, ultra-processed foods.

Among men, overweight/obesity prevalence was already elevated in donors aged < 35 years and further increased in older age groups across dietary patterns. Overall, these stratified analyses suggest that age-, sex-, and lifestyle-related differences coexist across dietary profiles, supporting the heterogeneity of the donor population.

### 3.7 Peri-donation dietary behaviors and self-reported dietary modifications

Overall, dietary changes were more frequently reported before donation than after donation (Table 5).

**TABLE 5:** Peri-donation dietary changes.

Period	Any change (%)	Decrease (%)	No change (%)	Increase (%)
Before donation	41.0	34.1	59.0	6.9
After donation	22.8	12.8	77.2	10.0

Values are expressed as percentages. “Any change” includes participants reporting either a decrease or an increase in intake compared with their usual diet.

**FIGURE 2:**
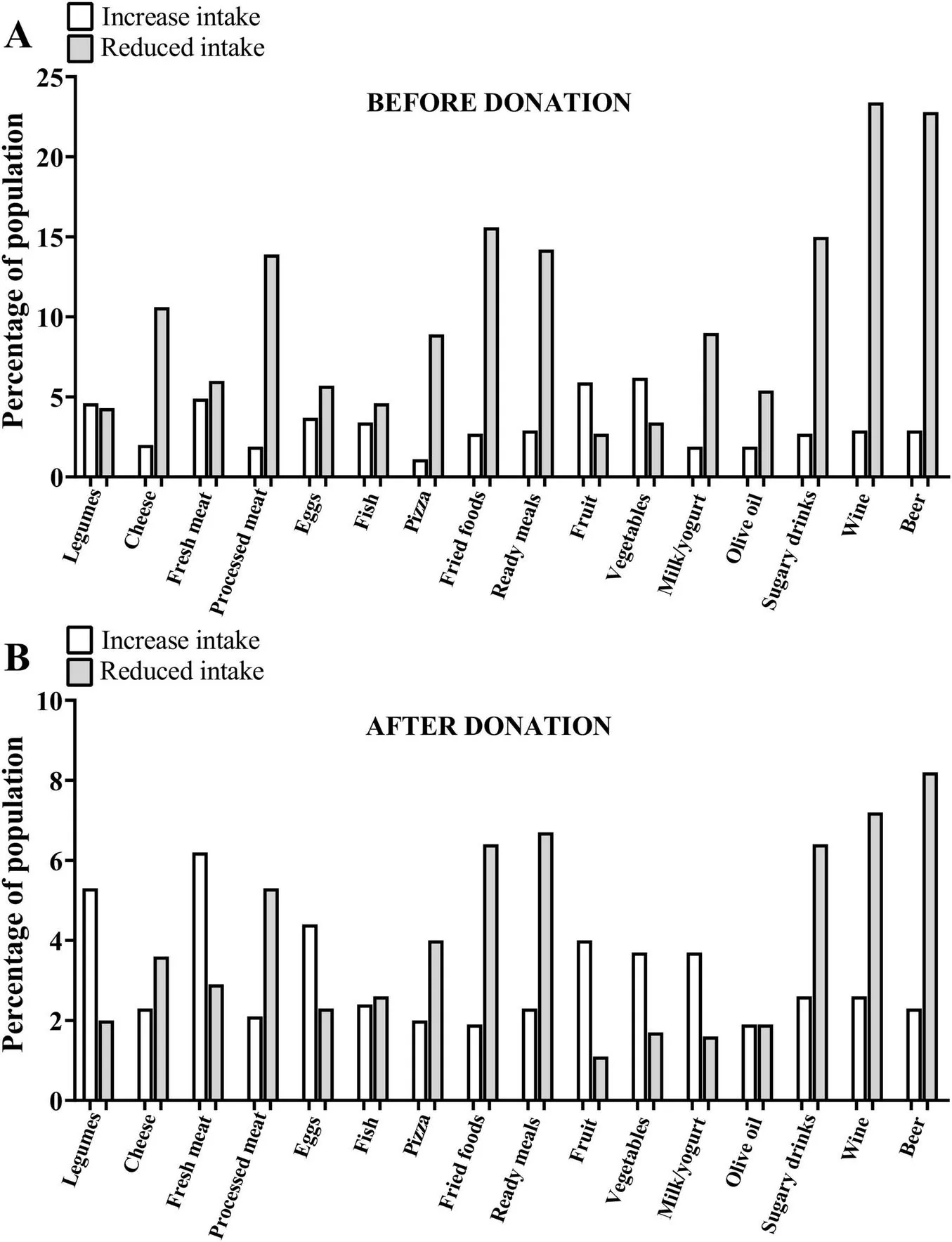
Self-reported dietary modifications **(A)** before and **(B)** after blood donation.

As shown in Figure 2, dietary modifications reported before donation were more frequent than those adopted after donation. Prior to donation (Figure 2A), a higher proportion of participants reported reducing the intake of foods perceived as potentially inappropriate before blood testing, particularly alcoholic beverages (wine and beer), sugary drinks, fried foods, ready meals, and processed meat.

After donation (Figure 2B), the overall percentage of participants reporting dietary changes markedly decreased. Although some respondents indicated increased consumption of iron-rich or nutrient-dense foods such as fresh meat, legumes, and vegetables, these percentages remained relatively low. Reductions in alcohol and high-fat foods persisted but at substantially lower levels compared with the pre-donation period.

### 3.8 Determinants of self-reported history of iron deficiency

In multivariable logistic regression analysis (Table 6), female sex emerged as the variable most strongly associated with self-reported history of iron deficiency (OR 4.62, 95% CI 3.20–6.68; *p* < 0.001). No significant associations were observed for age, BMI, smoking status, regular physical activity, or dietary pattern membership.

**TABLE 6:** Multivariable logistic regression for self-reported history of iron deficiency.

Response	Predictors	OR	95% CI		*p*-value
			Lower	Upper	
Self-reported history of iron deficiency	Female sex	4.62	3.20	6.68	< 0.001
	Age (per 10-year increase)	0.98	0.86	1.11	0.730
	BMI (per 1 kg/m^2^)	1.00	0.96	1.04	0.964
	Smoking (yes vs. no)	0.96	0.68	1.35	0.814
	Regular physical activity	0.88	0.63	1.22	0.441
	Cluster Mediterranean vs. Western	1.13	0.74	1.73	0.565
	Cluster Mixed vs. Western	1.20	0.84	1.71	0.316

Multivariable logistic regression model adjusted for age, sex, body mass index, smoking status, regular physical activity, and dietary pattern membership. Participants reporting either self-reported previous or recent iron deficiency were classified as positive for the outcome variable, whereas participants answering “do not know” were excluded from the analysis.

Notes. Multivariable logistic regression model adjusted for age, sex, body mass index, smoking status, physical activity, and dietary pattern membership. Participants reporting either self-reported previous or recent iron deficiency were classified as positive for the outcome variable, whereas participants answering “do not know” were excluded from the analysis.

### 3.9 Determinants of self-reported post-donation symptoms

Similarly, female sex was independently associated with a higher likelihood of self-reported post-donation symptoms (OR 3.07, 95% CI 2.09–4.52; *p* < 0.001), whereas increasing age was independently associated with a lower likelihood of symptom reporting (OR 0.73 per 10-year increase, 95% CI 0.64–0.84; *p* < 0.001) (Table 7). Lifestyle factors and dietary patterns were not significantly associated with self-reported post-donation symptoms.

**TABLE 7:** Multivariable logistic regression for self-reported post-donation symptoms.

Response	Predictors	OR	95% CI		*p-*value
			Lower	Upper	
Self-reported post-donation symptoms	Female sex	3.07	2.09	4.52	< 0.001
	Age (per 10-year increase)	0.73	0.64	0.84	< 0.001
	BMI (per 1 kg/m^2^)	1.01	0.97	1.04	0.715
	Smoking (yes vs. no)	1.28	0.91	1.80	0.158
	Regular physical activity	0.89	0.64	1.23	0.481
	Cluster Mediterranean vs. Western	1.24	0.79	1.94	0.349
	Cluster Mixed vs. Western	1.19	0.83	1.71	0.343

Multivariable logistic regression model adjusted for age, sex, body mass index, smoking status, regular physical activity, and dietary pattern membership.

Additional age-stratified logistic regression analyses confirmed progressively lower odds of self-reported post-donation symptoms across increasing age categories (Supplementary Table 5).

## 4 Discussion

Our study provides a comprehensive characterization of nutritional behaviors, lifestyle profiles, and donation-related outcomes within a large population of blood donors, highlighting important differences across donor subgroups. Although blood donors are generally considered healthy individuals because of routine eligibility procedures, our findings indicate that clinically relevant metabolic and behavioral vulnerabilities remain highly prevalent within donor populations. In particular, overweight and obesity affected nearly half of the cohort, while low physical activity levels, smoking habits, and suboptimal dietary behaviors were frequently observed despite continued donation eligibility. These findings are consistent with previous studies reporting substantial cardiometabolic risk burden among blood donors despite the well-recognized “healthy donor effect”(30–33). Important sex-, age-, and diet-related differences emerged across donor subgroups. Women more frequently reported self-reported history of iron deficiency and post-donation symptoms, findings consistent with previous hemovigilance studies identifying female sex as a major risk factor for iron depletion and donation-related adverse events (29, 34–36). Lower baseline iron stores, menstrual blood loss, lower circulating blood volume, and sex-related differences in cardiovascular and autonomic responses may contribute to this increased susceptibility. In contrast, men showed a less favorable metabolic and dietary profile characterized by a higher prevalence of overweight and obesity, lower fruit and vegetable intake, and greater consumption of processed meat, alcohol, sugar-sweetened beverages, and ultra-processed foods. Age-stratified analyses further highlighted different lifestyle and metabolic patterns across age groups. Younger donors more frequently showed less favorable dietary and lifestyle profiles, together with higher smoking prevalence, lower physical activity levels, and greater reporting of post-donation symptoms, whereas older donors showed more favorable dietary habits together with lower smoking prevalence, but also greater prevalence of overweight and obesity. Notably, overweight and obesity remained highly prevalent among men across all dietary profiles, including Mediterranean-like patterns, particularly among donors aged ≥ 35 years. Despite substantial differences across dietary and lifestyle profiles, dietary pattern membership was not independently associated with self-reported history of iron deficiency or post-donation symptoms in multivariable analyses. This finding likely reflects the multifactorial nature of donation-related outcomes. Iron homeostasis is influenced not only by dietary intake, but also by iron bioavailability, menstrual blood loss, inflammation, donation frequency, and interindividual variability in erythropoietic responses (16, 23, 26). Similarly, post-donation symptoms are influenced by multiple physiological and behavioral determinants, including hydration status, blood volume, autonomic responsiveness, and anxiety-related responses to donation (9–11). Accordingly, global dietary patterns alone may be insufficient to predict acute donation-related outcomes within a cross-sectional framework. Nevertheless, dietary profiles remained clinically informative because they reflected broader lifestyle-related profiles across donor subgroups.

Peri-donation dietary behaviors provided additional insights into donor perceptions regarding nutrition and donation safety. Dietary modifications were considerably more frequent before donation than after donation, with participants mainly reporting reductions in alcohol, fried foods, ready meals, and other foods perceived as potentially inappropriate before blood testing. In contrast, relatively few donors reported adopting recovery-oriented dietary behaviors after donation, despite the physiological demands associated with restoration of erythrocyte mass and iron stores following whole blood donation (6, 18–21). Consistently, although overall nutrition-related awareness appeared satisfactory, less accurate responses emerged for more specific aspects related to meal composition and nutrient interactions affecting iron absorption.

From a broader preventive perspective, these findings suggest that donor care should extend beyond procedural eligibility assessment alone and include greater attention to nutritional and lifestyle support. Female donors may particularly benefit from strategies focused on prevention and early recognition of iron depletion together with recovery-oriented nutritional recommendations after donation. Younger donors may require greater attention to unhealthy dietary patterns, smoking habits, and inadequate post-donation recovery behaviors, whereas adult and older donors may benefit from lifestyle-oriented interventions targeting overweight, obesity, and physical inactivity despite continued donation eligibility. Such approaches may help preserve donor health over time and potentially contribute to reducing donor attrition, a particularly relevant issue in transfusion systems increasingly dependent on regular donors. Nevertheless, their effectiveness should be confirmed in prospective longitudinal and interventional studies.

This study has several strengths. It included a large and heterogeneous donor population and simultaneously evaluated anthropometric characteristics, lifestyle behaviors, dietary intake, peri-donation practices, nutrition-related awareness, and donation-related outcomes within a unified analytical framework. The combined use of dietary pattern analysis, multivariable regression models, and age- and sex-stratified analyses allowed a more comprehensive characterization of donor heterogeneity than usually reported in donor-based nutritional surveys. To our knowledge, few studies have simultaneously explored dietary behaviors, nutrition-related awareness, peri-donation practices, and metabolic/lifestyle profiles within a large donor population.

Some limitations should nevertheless be acknowledged. The cross-sectional design precludes causal inference and limits temporal interpretation of the observed associations. Therefore, the observed relationships should be interpreted as associations only and cannot establish whether specific nutritional or lifestyle characteristics influence long-term donor health outcomes. Moreover, because multiple statistical comparisons were performed without formal correction, the possibility of type I error cannot be excluded.

In addition, all variables, including anthropometric measures, dietary intake, physical activity, self-reported history of iron deficiency, and post-donation symptoms, were self-reported and therefore potentially subject to recall bias, reporting bias, and misclassification. This may have affected the accuracy of prevalence estimates and attenuated or inflated some of the observed associations.

Although the questionnaire was developed using literature-informed item selection, multidisciplinary expert review, and pilot testing, it was not formally psychometrically validated as a whole. Construct validity and test–retest reproducibility were not assessed, although the six nutrition knowledge items showed acceptable internal consistency.

Dietary intake was assessed using semi-quantitative frequency categories rather than quantitative dietary assessment tools, preventing precise nutrient quantification. Information regarding donation frequency, cumulative lifetime donations, time since last donation, donation intensity, and iron supplementation was unavailable, limiting the evaluation of cumulative donation burden and its potential impact on iron status and post-donation tolerance.

In addition, sex-specific reproductive and hormonal variables, including menstrual blood loss, menopausal status, hormonal contraceptive use, and menopausal hormone therapy, were not collected. These factors may be particularly relevant when interpreting the higher likelihood of self-reported iron deficiency observed among female donors. Previous evidence has shown that menstrual blood loss may independently influence hemoglobin and ferritin levels in premenopausal blood donors (37), while menopausal status may further contribute to differences in iron-related risk profiles (18). Therefore, the association between female sex and self-reported iron deficiency should be interpreted with caution, as it may partly reflect unmeasured reproductive and hormonal determinants.

Moreover, objective biochemical markers of iron status, including serum ferritin, hemoglobin concentration, transferrin saturation, and other iron-related parameters, were unavailable. Therefore, self-reported history of iron deficiency could not be clinically validated, and the associations observed for this outcome should be interpreted with caution.

Finally, the web-based recruitment strategy, which relied on social media dissemination and institutional mailing lists, may have introduced selection bias by preferentially reaching younger, more health-conscious, or more digitally engaged donors. Therefore, the study population may not be fully representative of the broader blood donor population, and the generalizability of the findings should be interpreted with caution.

## 5 Conclusion

Overall, our findings highlight the importance of considering nutritional and lifestyle factors as integral components of modern donor care. Beyond procedural eligibility alone, greater attention to long-term metabolic health, dietary habits, and post-donation recovery may help support more comprehensive donor management strategies. Integrating nutritional counseling and lifestyle-oriented preventive approaches into donor care pathways may represent a promising strategy to support donor health; however, their effectiveness in preserving long-term donor health and supporting donor retention should be confirmed in future prospective and interventional studies.

## Data Availability

The raw data supporting the conclusions of this article will be made available by the authors, without undue reservation.
