# Supplementary material for: Beyond donor eligibility: nutritional and lifestyle heterogeneity and implications for long-term donor health preservation
Source: Front Nutr. 2026 Sep 3;13:1903623. doi: 10.3389/fnut.2026.1903623 (PMC13581511; doi:10.3389/fnut.2026.1903623)
Supplement: Supplementary file 1 [file Table_1.docx]

Supplementary Material

Table S1. Survey Questionnaire

Dear participant,We are conducting a survey on the dietary habits of blood donors. The research team belongs to the Clinical Nutrition Unit of the University Hospital of Catanzaro. Completion of this questionnaire is anonymous and requires only a few minutes. Please select the answer that best reflects your opinion or situation. Responses will remain confidential and will be used exclusively for statistical purposes in accordance with current data protection regulations. Thank you for your valuable cooperation.

Age (years):

__________________________________

Sex:

- Male
- Female

Marital status:

- Single
- Separated/divorced
- Married or cohabiting
- Widowed

What is your main occupation?

- Student
- Office work
- Manual/physical work
- Healthcare professional
- Unemployed

Does your work influence your dietary habits?

- No, I am able to follow a balanced diet
- Yes, I often eat outside the home or have quick meals
- Yes, irregular working hours affect my diet

Current body weight (kg):

________________________________________

Height (cm):

________________________________________

Do you smoke?

- Yes
- No

Do you engage in physical activity?

- Yes
- Yes, occasionally
- No

Have you ever experienced iron deficiency?

- Yes, currently
- Yes, in the past
- No, never
- I do not know

Have you been diagnosed with one or more of the following conditions?

- Rheumatoid arthritis or other forms of arthritis
- Systemic lupus erythematosus, systemic sclerosis, Sjögren’s syndrome, or other connective tissue diseases
- Anxiety
- Depression
- Diabetes
- Dyslipidemia (hypercholesterolemia and/or hypertriglyceridemia)
- Arterial hypertension
- Osteoporosis
- Chronic respiratory diseases (e.g., COPD, emphysema, asthma)
- Previous myocardial infarction
- Previous stroke
- Other chronic neurological diseases (e.g., epilepsy, Parkinson’s disease, multiple sclerosis)
- Solid or hematological malignancies (e.g., leukemia or lymphoma)
- None

Do you regularly take medications?

- Yes, occasionally (e.g., painkillers)
- Yes, for chronic diseases
- No

How much salt do you consume daily?

- More than 5 g
- Less than 5 g
- Approximately 5 g
- I do not know

Weekly Food Frequency (number of portions per week: none, 1, 2, 3, or more)

*Standard portion sizes:*
50 g dried legumes; 150 g fresh legumes; 100 g fresh cheese; 50 g aged cheese; 100 g fresh meat; 50 g processed meat; 2 eggs; 150 g fish.

- Legumes
- Fresh and/or aged cheese
- Fresh meat
- Processed meats/cold cuts
- Eggs
- Fresh or frozen fish
- Canned tuna

Weekly consumption (none, 1, 2, 3, or more portions)

- Pizza
- Flour for homemade recipes (pizza, fresh pasta, bread, cakes, biscuits, etc.)
- Fried foods
- Ready-to-eat meals (frozen foods, fast food)
- Biscuits or snack cakes
- Sausages (wieners)
- Chips and packaged snacks

Alcohol consumption (glasses per week: none, 1–2, 3–4, ≥5)

- Wine
- Beer
- Spirits
- Alcoholic aperitifs
- Digestive liqueurs

Daily Food Frequency (none, <1, 1, 2, 3, or more portions)

*Standard portion sizes:*
125 mL/g (milk/yogurt); oils 10 g (1 tablespoon); jam 10 g; margarine 10 g; butter 10 g; coffee (1 cup); bread 30 g; beverages 200 mL.

- Milk and/or yogurt
- Sugar
- Jam
- Breakfast cereals
- Bread
- Pasta or rice
- Coffee
- Vegetables
- Fresh fruit
- Nuts
- Olive oil
- Seed oils
- Butter
- Margarine
- Chocolate
- Ice cream and filled desserts
- Biscuits and homemade sweets
- Sugar-sweetened beverages

Dietary Pattern

Do you follow a specific diet?

- Vegetarian
- Vegan
- Pescatarian
- No specific diet (omnivorous)
- Other

Are your eating habits influenced by blood donation?

- Yes
- No, I eat normally
- No, I have never considered it

Compared with your usual diet, during the week BEFORE donation do you reduce, maintain, or increase your intake?

- Milk and/or yogurt
- Sugar
- Jam
- Breakfast cereals
- Bread
- Pasta or rice
- Coffee
- Vegetables
- Fresh fruit
- Nuts
- Olive oil
- Seed oil
- Butter
- Margarine
- Chocolate
- Ice cream and filled desserts
- Biscuits and homemade sweets
- Sugar-sweetened beverages
- Legumes
- Fresh and/or aged cheese
- Processed meats and cold cuts
- Fresh meat
- Eggs
- Fresh and frozen fish
- Canned tuna
- Pizza
- Flour for homemade recipes
- Fried foods
- Ready meals
- Wine
- Beer
- Spirits
- Alcoholic aperitif
- Digestive liqueurs

Compared with your usual diet, during the week AFTER donation do you reduce, maintain, or increase your intake?

- Milk and/or yogurt
- Sugar
- Jam
- Breakfast cereals
- Bread
- Pasta or rice
- Coffee
- Vegetables
- Fresh fruit
- Nuts
- Olive oil
- Seed oil
- Butter
- Margarine
- Chocolate
- Ice cream and filled desserts
- Biscuits and homemade sweets
- Sugar-sweetened beverages
- Legumes
- Fresh and/or aged cheese
- Processed meats and cold cuts
- Fresh meat
- Eggs
- Fresh and frozen fish
- Canned tuna
- Pizza
- Flour for homemade recipes
- Fried foods
- Ready meals
- Wine
- Beer
- Spirits
- Alcoholic aperitif
- Digestive liqueurs

Did you know that a high-fat meal before donation may alter blood test results?

- Yes
- No

Do you know that some vitamins are essential for red blood cell synthesis?

- Yes
- No

Do you know that it is recommended to drink plenty of water before and after donation?

- Yes
- No

Did you know that some foods may increase or decrease iron absorption?

- Yes
- No

Do you know that vitamin C helps iron absorption?

- Yes
- No

Do you know that tea and coffee reduce iron absorption if consumed during meals?

- Yes
- No

Total cholesterol at your last medical check-up:

- <200 mg/dL
- 200–239 mg/dL
- 240 mg/dL

Serum iron (sideremia) at your last medical check-up:

- Below 50 µg/dL (women) / below 65 µg/dL (men)
- Between 50 and 170 µg/dL (women) / between 65 and 176 µg/dL (men)
- Above 150 µg/dL (women) / above 176 µg/dL (men)

Hemoglobin value at your last medical check-up:

- Below 12 g/dL (women) / 13 g/dL (men)
- Between 12–15 g/dL (women) / 13–17 g/dL (men)
- Above 15 g/dL (women) / 17 g/dL (men)

Where did you receive information about nutrition for donation?

- Physician/AVIS
- Internet
- Friends or relatives

Would you like to receive more information on what to eat before and after donation?

- Yes
- No
- Indifferent

Which nutritional information would you like to explore further?

- Diet to prepare for donation
- Strategies to prevent iron and vitamin deficiencies
- Better post-donation recovery habits

Which channel would you prefer to receive this information?

- Brochure
- Educational videos
- Events or meetings with experts
- Social media

After donation

Did you experience any particular symptoms after donation?

- No
- Yes, severe symptoms (dizziness, severe arm pain, palpitations, nausea)
- Yes, common symptoms (fatigue, weakness)

Have you changed your eating habits since donating blood?

- Yes, I started following a more balanced diet
- Yes, I reduced unhealthy foods
- No, my eating habits remained the same
- No, but I would like to improve my diet

**Table S2.** Demographic and clinical characteristics by age group

| **Variable** | **18–29** | **30–44** | **45–59** | **≥60** | **p-value** |
| --- | --- | --- | --- | --- | --- |
| Female, n (%) | 230 (69.1) | 77 (47.0) | 80 (49.7) | 14 (35.0) | <0.001 |
| BMI, kg/m² | 24.2 ± 4.0 | 25.6 ± 4.2 | 26.8 ± 4.4 | 27.4 ± 4.1 | <0.001 |
| Overweight/obesity (BMI ≥25), n (%) | 114 (34.2) | 80 (48.8) | 108 (67.1) | 28 (70.0) | <0.001 |
| Smoking, n (%) | 98 (29.4) | 50 (30.5) | 38 (23.6) | 6 (15.0) | 0.126 |
| Regular physical activity, n (%) | 153 (45.9) | 61 (37.2) | 45 (28.0) | 10 (25.0) | <0.001 |
| Iron deficiency, n (%) | 121 (36.3) | 50 (30.5) | 58 (36.0) | 15 (37.5) | 0.590 |
| Post-donation symptoms, n (%) | 153 (45.9) | 47 (28.7) | 37 (23.0) | 5 (12.5) | <0.001 |

Notes. Continuous variables are presented as mean ± standard deviation (SD) and were compared using one-way analysis of variance (ANOVA). Categorical variables are expressed as n (%) and were compared using chi-square (χ²) tests. BMI: Body Mass Index; ID: Iron Deficiency.

**Table S3.** Detailed habitual dietary intake stratified by age group (portions/day or week)

| **Food group** | **18–29** | **30–44** | **45–59** | **≥60** | **p-value** |
| --- | --- | --- | --- | --- | --- |
| Legumes (week) | 1.38 ± 0.92 | 1.43 ± 0.83 | 1.28 ± 0.72 | 1.50 ± 0.72 | 0.296 |
| Cheese (week) | 1.65 ± 0.96 | 1.59 ± 0.83 | 1.59 ± 0.83 | 1.80 ± 0.94 | 0.535 |
| Fresh meat (week) | 2.04 ± 0.90 | 1.89 ± 0.87 | 1.55 ± 0.81 | 1.43 ± 0.68 | <0.001 |
| Processed meat (week) | 1.25 ± 0.91 | 1.13 ± 0.82 | 1.17 ± 0.90 | 1.27 ± 0.91 | 0.514 |
| Eggs (week) | 1.42 ± 0.82 | 1.44 ± 0.77 | 1.40 ± 0.64 | 1.18 ± 0.87 | 0.265 |
| Fish (week) | 1.51 ± 0.79 | 1.60 ± 0.82 | 1.70 ± 0.82 | 1.78 ± 0.83 | 0.004 |
| Pizza (week) | 1.62 ± 0.93 | 1.41 ± 0.89 | 1.16 ± 0.82 | 0.98 ± 0.77 | <0.001 |
| Fried foods (week) | 1.45 ± 0.91 | 1.37 ± 0.86 | 1.29 ± 0.80 | 1.15 ± 0.77 | 0.013 |
| Ready meals (week) | 1.51 ± 0.89 | 1.40 ± 0.88 | 1.26 ± 0.80 | 1.20 ± 0.76 | 0.003 |
| Fruit (day) | 1.58 ± 0.94 | 1.64 ± 0.95 | 1.77 ± 0.94 | 1.90 ± 1.00 | 0.020 |
| Vegetables (day) | 1.72 ± 0.95 | 1.82 ± 0.94 | 1.95 ± 0.96 | 2.03 ± 0.97 | 0.010 |
| Milk/yogurt (day) | 1.49 ± 0.91 | 1.45 ± 0.90 | 1.44 ± 0.88 | 1.46 ± 0.95 | 0.920 |
| Olive oil (day) | 1.53 ± 0.82 | 1.59 ± 0.81 | 1.67 ± 0.82 | 1.73 ± 0.85 | 0.030 |
| Sugary drinks (day) | 1.45 ± 0.91 | 1.37 ± 0.85 | 1.28 ± 0.80 | 1.15 ± 0.78 | 0.009 |
| Wine (week) | 1.22 ± 0.85 | 1.52 ± 0.93 | 1.63 ± 0.95 | 1.60 ± 0.95 | <0.001 |
| Beer (week) | 1.48 ± 0.87 | 1.42 ± 0.86 | 1.26 ± 0.82 | 1.15 ± 0.80 | 0.001 |

Notes. Food frequency responses were coded as ordinal numeric variables (0–3) as described above. Continuous variables are expressed as mean ± SD and were compared using one-way ANOVA.

**Table S4.** Rotated factor loadings from principal component analysis (PCA) of habitual dietary intake variables

| **Food group** | **Mediterranean-like** | | **Western/ultra-processed-like** | | **Mixed beverage-oriented pattern** |
| --- | --- | --- | --- | --- | --- |
| Vegetables | **0.809** | | -0.116 | | -0.009 |
| Fruit | **0.775** | | -0.063 | | -0.007 |
| Olive oil | **0.699** | | 0.182 | | -0.055 |
| Milk/yogurt | **0.586** | | 0.040 | | -0.141 |
| Legumes | **0.411** | | -0.287 | | 0.186 |
| Fish | **0.420** | | -0.111 | | **0.321** |
| Eggs | **0.403** | | -0.059 | | **0.356** |
| Processed meat | -0.063 | | **0.690** | | 0.077 |
| Fried foods | -0.086 | | **0.629** | | 0.201 |
| Ready meals | -0.036 | | **0.615** | | -0.143 |
| Sugary drinks | -0.003 | | **0.560** | | -0.021 |
| Pizza | -0.034 | | **0.507** | | 0.091 |
| Fresh meat | 0.176 | | **0.416** | | 0.158 |
| Cheese | 0.171 | | **0.338** | | 0.122 |
| Wine | -0.012 | | -0.022 | | **0.804** |
| Beer | -0.096 | | 0.198 | | **0.705** |
| **Variance explained by each component** | | | | | |
| **Component** | | **Explained variance (%)** | | **Cumulative variance (%)** | |
| Mediterranean-like | | 17.3 | | 17.3 | |
| Western/ultra-processed-like | | 14.4 | | 31.7 | |
| Mixed beverage-oriented pattern | | 9.0 | | 40.7 | |

Note. PCA, principal component analysis. Dietary intake variables were standardized prior to analysis. Varimax rotation was applied to improve interpretability. Factor loadings represent correlations between food groups and dietary patterns. Loadings ≥ |0.30| were considered meaningful.

**Table S5.** Additional and sensitivity logistic regression analyses

| **Predictor** | **Symptoms (age-stratified) OR (95% CI)** | **Iron deficiency (cluster-adjusted) OR (95% CI)** | **Symptoms (cluster-adjusted) OR (95% CI)** |
| --- | --- | --- | --- |
| Female sex | 3.05 (2.07–4.50)*** | 4.63 (3.20–6.70)*** | 3.07 (2.09–4.52)*** |
| Age 30–44 vs 18–29 | 0.49 (0.33–0.73)*** | — | — |
| Age 45–59 vs 18–29 | 0.36 (0.24–0.55)*** | — | — |
| Age ≥60 vs 18–29 | 0.18 (0.07–0.45)*** | — | — |
| Age (per 10 y) | — | 0.98 (0.86–1.12) | 0.73 (0.64–0.84)*** |
| BMI (per kg/m²) | 1.01 (0.97–1.04) | 1.00 (0.96–1.04) | 1.01 (0.97–1.04) |
| Smoking | 1.29 (0.91–1.82) | 0.95 (0.67–1.34) | 1.28 (0.91–1.81) |
| Physical activity | 0.90 (0.64–1.25) | 0.88 (0.63–1.23) | 0.89 (0.64–1.24) |
| Mediterranean vs Western | — | 1.13 (0.74–1.73) | 1.24 (0.79–1.94) |
| Mixed vs Western | — | 1.20 (0.84–1.71) | 1.19 (0.83–1.71) |

Notes. ***p < 0.001; Multivariable logistic regression models were adjusted for sex, age, BMI, smoking status, physical activity, and dietary pattern membership. Cluster-adjusted models additionally included dietary pattern membership. Odds ratios (ORs) and 95% confidence intervals (CIs) are reported. Reference categories: male sex, age 18–29 years, and Western dietary cluster.
